# Identification, characterization and molecular analysis of the viable but nonculturable *Rhodococcus biphenylivorans*

**DOI:** 10.1038/srep18590

**Published:** 2015-12-21

**Authors:** Xiaomei Su, Faqian Sun, Yalin Wang, Muhammad Zaffar Hashmi, Li Guo, Linxian Ding, Chaofeng Shen

**Affiliations:** 1 Department of Environmental Engineering, College of Environmental and Resource Sciences, Zhejiang University, Hangzhou 310058, China; 2 Key Laboratory for Water Pollution Control and Environmental Safety, Zhejiang Province, China; 3 Department of Meteorology, COMSATS Institute of Information Technology, Islamabad 44000, Pakistan; 4 College of Geography and Environmental Science, Zhejiang Normal University, Jinhua 321004, China

## Abstract

Numerous bacteria, including pollutant-degrading bacteria can enter the viable but nonculturable state (VBNC) when they encounter harsh environmental conditions. VBNC bacteria, as a vast majority of potent microbial resource can be of great significance in environmental rehabilitation. It is necessary to study the VBNC state of pollutant-degrading bacteria under various stress conditions. The aim of this study was to determine whether *Rhodococcus biphenylivorans* could enter the VBNC state under oligotrophic and low temperature conditions, and to examine the changes of morphology, enzymatic activity and gene expressions that might underline such state. The obtained results indicated that *R*. *biphenylivorans* TG9^T^ could enter into the VBNC state and recover culturability under favorable environmental conditions. Results from Illumina high throughput RNA-sequencing revealed that the up-regulated genes related to ATP accumulation, protein modification, peptidoglycan biosynthesis and RNA polymerase were found in the VBNC cells, and the down-regulated genes mainly encoded hypothetical protein, membrane protein and NADH dehydrogenase subunit, which render VBNC cells more tolerant to survive under inhospitable conditions. This study provides new insights into prevention and control of the VBNC state of pollutant-degrading bacteria for their better capabilities in environmental rehabilitation.

Since the pioneering studies of Xu *et al.*^1^, much information on the survival strategy of non-sporulating bacteria in adverse environment stresses has accumulated indicating that bacteria can enter a viable but nonculturable state (VBNC) when they encounter harsh environmental conditions^2^,^3^. In such a state, bacteria are still alive and retain metabolic activity but fail to form colonies on conventional bacteriological media. In addition, cells that enter the VBNC state exhibit major morphological and metabolic modifications, including reduction in size, decreased metabolic activity, alterations in cell membrane composition and cell wall structure^4^,^5^. Generally, the cells of rod-shaped bacteria shrink in size, and decrease in macromolecules synthesis, respiration rate as well as nutrient transport when enter into the VBNC state. By contrast, membrane potential and ATP levels remain high^6^. VBNC bacteria could recover their culturability when existing conditions become more favorable^2^,^7^. In addition, resuscitation of VBNC bacteria may involve scout cells that sample the environment for reproductive suitability^5^,^7^.

In natural environments, it is very common for bacteria to survive by entering the VBNC state in response to a wide variety of harsh conditions including extreme temperature, osmotic concentrations, oligotrophic nutrients, oxygen, copper stress and organic pollutants^8^,^9^. Up to now, over 60 bacterial species have been verified to enter into the VBNC state. Most of studies focused on VBNC bacteria due to their infectious and pathogenic potentials which are closely related to human, animal and plant health^3^,^6^,^9^,^10^,^11^. Meanwhile, several methods have been developed to check the presence of VBNC bacteria, such as checking membrane integrity, enzymatic activity and respiration rate^12^,^13^. A combination of flow cytometry (FCM) with SYTO 9/propidium iodide (PI) and conventional cultivation-based techniques was used to count viable and culturable cells for confirming viability in nonculturable bacteria^14^. ATP assay enzyme mix solution was used to measure the change of intracellular ATP levels between VBNC cells and normal cells^6^,^15^.

In recent years, gene expression and the proteins modulation of VBNC cells have been widely studied^16^,^17^,^18^. Generally, real-time PCR-based approach was used to investigate known genes involved in response to stress-provoking conditions, while RNA transcription was employed to assess the gene expression in the VBNC state^5^,^17^,^19^. In a recent report, the expression of *rpoS* and *relA* genes was found relatively high in the VBNC state compared with the starved cells of *Vibrio cholerae* by using quantitative real-time reverse transcription PCR (Q-RT-PCR)^17^. Remarkably, the sigma factor encoded by *rpo*S gene is essential for survival of numerous gram-negative bacteria under multiple stress conditions, because the sigma factor RpoS regulates expression of several proteins such as cyclopropane-fatty-acyl-phospholipid synthase and catalase^19^. Transcriptional control of *rpo*S involves the accumulation of guanosine 3’,5’-bispyrophosphate (ppGpp) which depends upon the activity of two proteins RelA and SpoT encoding by *rel*A and *spo*T genes, respectively^20^. Evidence has been obtained that the *rpo*S expression is enhanced as a result of the elevated concentration of ppGpp, which ultimately enhance the capability of bacterial stress resistance^5^,^20^. In addition, Mathur *et al.*^21^ found that a major *V*. *cholerae* outer membrane protein, OmpU protein acts as a signal in the activation of RpoE which is an alternative sigma involving the membrane response.

Although a large amount of work has been done characterizing the formation and resuscitation of the VBNC state in pathogenic bacteria^2^,^6^,^18^,^21^, at present there is hardly any information regarding the VBNC state in pollutant-degrading bacteria. In fact, most bacteria in natural environments cannot be cultivated, and just over 7000 well-founded species have been described to date^22^. It is worth pointing out that VBNC bacteria in the polluted environments can be of great significance in environmental rehabilitation, since VBNC bacteria represent a vast majority of potent microbial resource. Therefore, studies are needed to elucidate the potentially environmental functions of VBNC bacteria, rather than only assess their role as potential pathogens from the view of epidemiology and public health. In our previous studies, we explored the uncultured or VBNC bacteria in the polychlorinated biphenyls (PCBs) contaminated environments by adding extracellular organic matter (EOM) from *Micrococcus luteus*^23^. Notably, the type strain TG9^T^ was isolated from the PCB-contaminated river sediment and the name *Rhodococcus biphenylivorans* sp. nov. was proposed^24^. Hence, study on the VBNC state of *R*. *biphenylivorans* in response to various stresses will provide a new insight for assessing the derivative activity of functional bacteria in natural environments. Moreover, a broader understanding of VBNC bacteria could help us reveal why highly efficient pollutant-degrading bacteria generally present lower activities in a pilot-scale environmental bioremediation.

In the present study, we aimed to investigate whether *R. biphenylivorans* could enter the VBNC state under oligotrophic and low temperature conditions, and under what conditions could resuscitate the VBNC cells. Moreover, the changes of morphology and enzymatic activity between the VBNC cells and normal cells were investigated. Specially, Illumina high throughput RNA-sequencing (RNA-Seq) was employed to identify differential gene expression at the stages of VBNC formation, and a relatively comprehensive understanding of the gene expression and regulation underlying such state was obtained. To the best of our knowledge, for the first time we comprehensively investigated the VBNC state of the biphenyl/PCB-degrading bacterium. Above all, the results would be helpful to provide substantial insight into enhancing the activities and degrading capabilities of pollutant-degrading bacteria. Likewise, prevention and control of VBNC bacteria are likely to be useful for improving their degradation function in a field-scale environmental bioremediation.

## Results

### Evidence for entering the VBNC state

**Figure 1:**
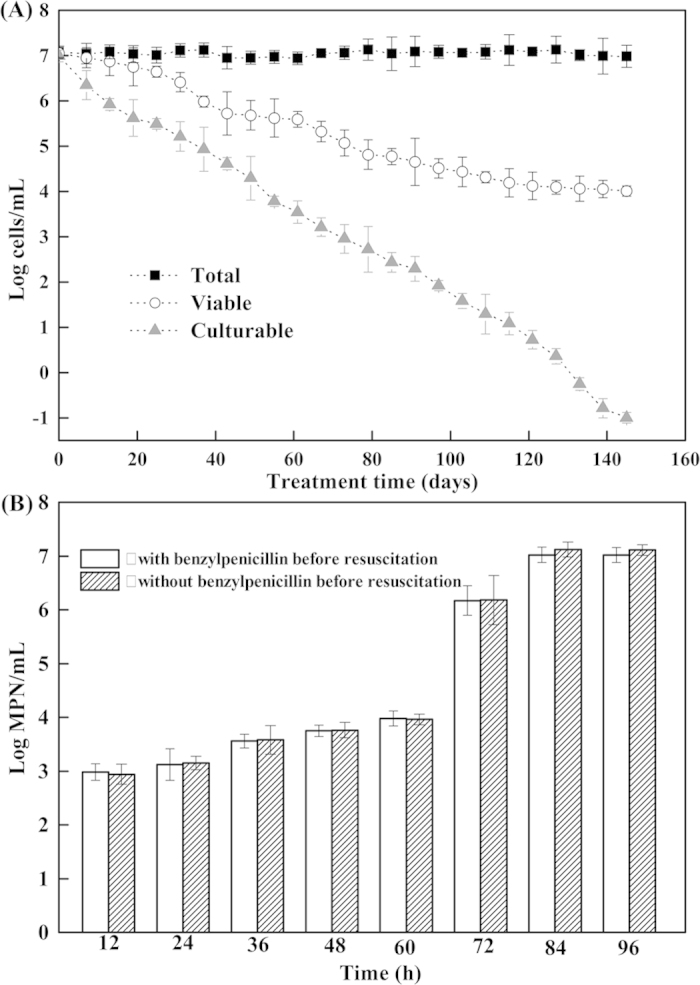
Evidence for entry of *Rhodococcus biphenylivorans* into the VBNC state. (**A**) entry into a VBNC state in a mineral-salts medium at 4 °C, as determined by AODC (⌜), FCM (○) and plate counting (▴) methods. (**B**) resuscitation of the VBNC cells. Error bars show the standard deviations of triplicate experiments.

Viabilities of exponential-phase cells (c_TG9) and VBNC cells (t_TG9) were investigated once a week during an experimental period of 5 months. As shown in Fig. 1A, the total number of cells remained constant at the initial level (10^7^ cells/mL), but the viable and culturable cells declined gradually. The number of culturable cells counted by visible colonies decreased to undetectable levels (<0.1 CFU/mL) after 145 days. However, the number of viable cells showed a low decrease (approximated 10^4^ cells/mL after 145 days), revealing that these cells were always alive. These results indicated that about 10^4^ cells/mL entered into the VBNC state under the present conditions. Moreover, the presence of VBNC cells was also verified by resuscitation.

Resuscitation experiments were performed in the VBNC microcosms by using two approaches of solid and liquid media. With solid media, colonies appeared when plating the cells on the Luria-Bertani (LB) agar plates at 30 °C for 3 days. With liquid media (Fig. 1B), when the incubation time was prolonged to 60 h, the most probable number (MPN) of the resuscitable cells rose to 9.55 × 10^3^ MPNs/mL. The value tended to increase rapidly between 60 h and 84 h, and reached a peak value (1.05 × 10^7^ MPNs/mL) at 84 h. These results indicated that the VBNC cells after 84 h resuscitation were in the stationary phase which was caused by the multiplication of the resuscitated cells. It is worth noting that the group treated with benzylpenicillin before resuscitation displayed no significant difference from the other group without benzylpenicillin pretreatment.

### Characterization of VBNC cells

**Figure 2:**
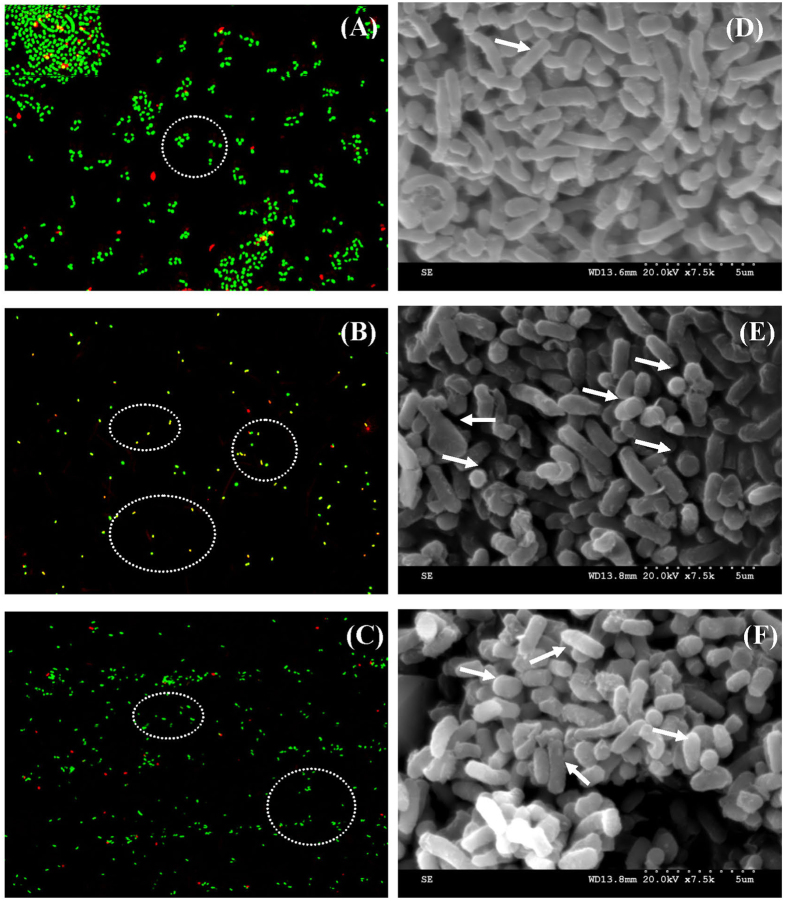
Morphological characteristics of *Rhodococcus biphenylivorans* under a confocal laser scanning microscope or a scanning electron microscopy. CLSM (**A–C**), SEM (**D–F**), exponential-phase cells (**A,D**), VBNC cells (**B,E**), resuscitated cells (**C,F**).

Significant morphological changes were observed between the exponential-phase cells and VBNC cells by using CLSM and SEM. After entering the VBNC state, the cells were significantly smaller than exponential-phase cells, and the fluorescence intensities become smaller indicating the presence of some double-staining cells (Fig. 2A,B, oval). Meanwhile, most of cells changed from rods to short rods or cocci and exhibited irregular cell morphology (Fig. 2D,E, arrow). Notably, the average size of the exponential-phase cells was 1.4 × 0.5 μm (length × width), while the VBNC cells was approximately 0.6 × 0.4 μm. Meanwhile, CLSM and SEM were also used to visualize the morphology of resuscitated cells in 84 h (Fig. 2C,F, oval and arrow). The resuscitated cells showed morphological differences compared with exponential-phase and VBNC cells. The average size of the resuscitated cells was 1.0 × 0.5 μm which is smaller than exponential-phase cells but larger than VBNC cells. The fluorescence intensity of resuscitated cells became stronger than VBNC cells, but weaker than exponential-phase cells. The results were consistent with the available data on MPNs of resuscitated cells, which indicated that the resuscitated cells had already multiplied and were in the stationary phase after 84 h resuscitation.

The cellular enzyme activities were assessed by using API ZYM kit according to the color intensity of each enzyme. As shown in Fig. S2, the activity of esterase (C4), esterase lipase (C8), leucine arylamidase, valine arylamidase, acid phosphatase, naphthol-AS-BI-phosphohydrolase, *α*-glucosidase and *β*-glucosidase were all identified in the three different cell states. These enzymes in the VBNC cells exhibited lower activities in comparison with those in exponential-phase cells. However, the enzymes of *α*-chymotrypsin and *β*-galactosidase, which were negative in the exponential-phase cells, demonstrated very weak activity in the VBNC cells. And the activity of the two enzymes was enhanced in the resuscitated cells.

### Overview of the transcriptome analysis in VBNC-response genes

cDNA libraries derived from the c_TG9 and t_TG9 were constructed, sequenced and generated with 13,438,262 and 12,843,362 reads, respectively. The sequence reads were aligned to the reference genome of *R*. *pyridinivorans* strain SB3094. Of the total reads, about 87.30% and 80.94% reads obtained from exponential-phase and VBNC samples, respectively, were covered in the genome database (Table 1). Within the mapped reads, approximately 72.54% and 70.12% of total reads were perfectly mapped to the reference genome without mismatch, respectively. Furthermore, each group covered more than 50% reads mapped to genes of the reference strain (Table 1). The numbers of perfectly matched reads and reads mapped to the annotated genes indicated that the obtained sequences met the requirement for further analysis.

Gene expression levels were determined by the average values of RPKM (reads per kilobase transcriptome per million mapped reads) obtained from biological replicate samples. The results revealed that 3768 and 3773 predicted genes were expressed in t_TG9 and c_TG9, respectively (Table S2). Notably, all genes in the t_TG9 were expressed with RPKM > 1, and 48.9% (1845 genes) of the expressed genes were detected with high expression values (RPKM > 100). However, 25 genes in the c_TG9 were expressed with 0 < RPKM ≤ 1, and 34.2% (1289 genes) of the expressed genes were detected with high expression values (RPKM > 100).

**Figure 3:**
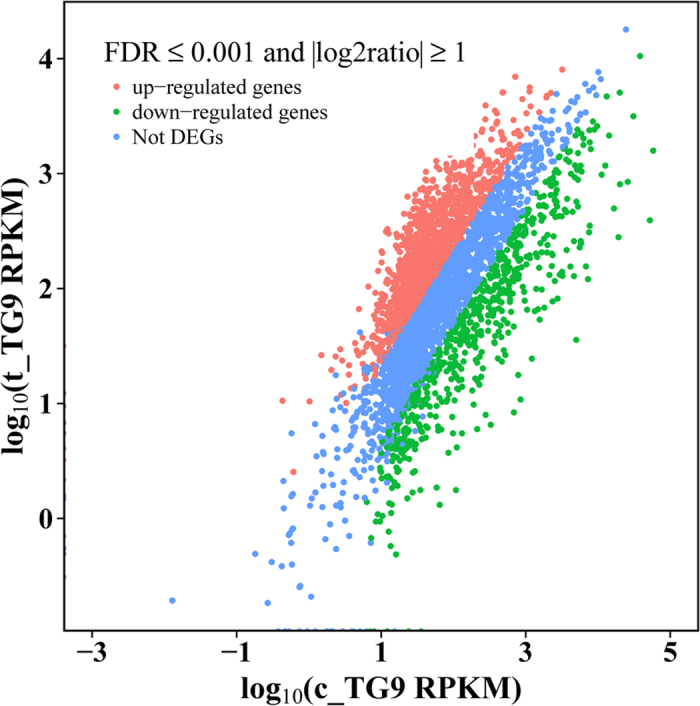
Differential expression level of t_TG9 versus c_TG9 using FDR ≤ 0.001 and |log2 ratio| ≥ 1.

To determine which genes were differentially expressed during the VBNC state formation, a false discovery rate (FDR) ≤ 0.001 and an absolute value of the log2 ratio (|log2 ratio|) ≥ 1 were used to evaluate the differential gene expression between t_TG9 and c_TG9 libraries. As shown in Fig. 3 and Table S3, a total of 2097 differentially expressed genes (DEGs) were detected, of which 1452 were up-regulated and 645 were down-regulated in the t_TG9 versus c_TG9 cultures. Those genes with expression fold change of at least 20-fold up or down-regulation are shown in Table S4. This analysis yielded a total of 59 DEGs, 17 of which were up-regulated and 42 down-regulated (Table S4).

### Gene Ontology (GO) functional analysis of DEGs

**Figure 4:**
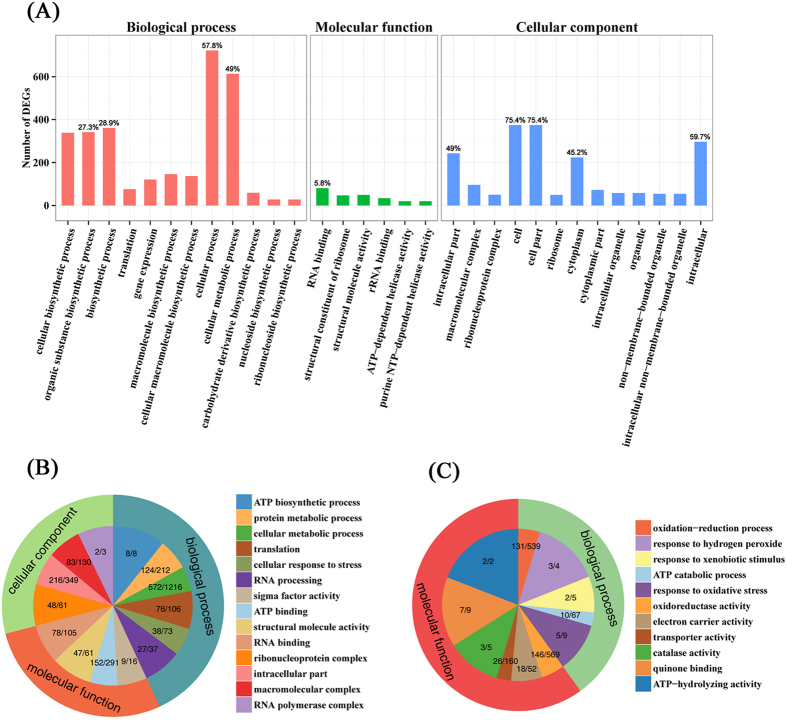
GO enrichment of differentially expressed genes. (**A**) The y-axis denotes the number of genes in a category. The number above the bar denotes the percentage of a specific term of genes in the main category. (**B**) GO enrichment of up-regulated genes. (**C**) GO enrichment of down-regulated genes. The significant enriched GO terms were presented with P-value < 0.05.

To ascribe gene functions to those DEGs, GO enrichment analysis was performed with the total of 2097 DEGs. As shown in Table S5, DEGs were assigned to at least one of the GO codes and categorized into 1357 secondary level GO terms, of which 786 were enriched in biological process, 504 were in molecular function, and 67 were in cellular component. The significantly enriched terms (*p*-value < 0.05) were indicated in Fig. 4A and Table S5 (in bold). In the 31 GO terms of significant enrichment, the DEGs involving in the three main categories of biological process (12 GO terms), molecular function (6 GO terms) and cellular component (13 GO terms) were 1250, 496 and 1385, respectively. In the 12 GO terms of biological process category, “cellular process” (722, 57.8%) and “cellular metabolic process” (613, 49.0%) were the most highly represented, followed by “biosynthetic process” (361, 28.9%) and “organic substance biosynthetic process” (341, 27.3%). Moreover, “RNA binding” (81, 5.8%) was dominant in molecular function category. In addition, in the cellular component category, a large number of DEGs were enriched in the “cell” (374, 75.4%), “cell part” (374, 75.4%), “intracellular” (296, 59.7%), “intracellular part” (243, 49.0%) and “cytoplasm” (224, 45.2%).

To further understand GO function of the up- or down-regulated genes, GO enrichment analysis was performed on the 1452 up-regulated and 645 down-regulated genes, respectively. As shown in Fig. 4B and Table S6, for the 2024 enrichment GO terms of up-regulated genes, there were 102, 38, and 18 significantly enriched (corrected *p*-value < 0.05, in bold) GO terms in each of the three main categories (biological process, molecular function, and cellular component), respectively. Specially, many up-regulated genes were significantly enriched in the category of biological process including “ATP biosynthetic process”, “protein metabolic process”, “cellular metabolic process”, “RNA processing” and “translation”. They were also significantly enriched in the category of molecular function including “ATP binding”, “structural molecule activity” and “RNA binding” (Fig. 4B, Table S6). In the category of cellular component, up-regulated genes were significantly enriched with the terms of “ribonucleoprotein complex”, “intracellular part” and “macromolecular complex”. Notably, the non-significant GO terms of “cellular response to stress”, “sigma factor activity” and “RNA polymerase complex” were main functions of up-regulated genes when compared up-regulated genes with the genome genes in each term. Interestingly, for the 1031 enrichment GO terms of down-regulated genes, there were only 9 terms significantly enriched (corrected *p*-value < 0.05, in bold) (Table S7), of which 1 and 8 GO terms belonged to the categories of biological process and molecular function, respectively. As shown in Table S7 and Fig. 4C, although the function of down-regulated genes covered a relatively comprehensive range of GO terms, they were mainly involved in “oxidation-reduction process”, “oxidoreductase activity”, “transporter activity” and “ATP catabolic process”. Furthermore, when compared down-regulated genes with the genome genes in each term, the terms of “response to hydrogen peroxide”, “response to xenobiotic stimulus”, “response to oxidative stress”, “catalase activity”, “quinone binding” and “ATP-hydrolyzing activity” were main functions of down-regulated genes.

### Pathway analysis of DEGs

To search for the DEGs involved in the significantly enriched metabolic pathways or signal transduction pathways, all of the genes were mapped to the Kyoto Encyclopedia of Genes and Genomes (KEGG) database and compared with the whole genomic background. The enrichment analysis results indicated that among all the 3307 genes with the KEGG pathway annotation, 1508 DEGs were involved in 174 pathways (Table S8). The pathways with the most representation were “metabolic pathways” (689, 45.7%), “biosynthesis of secondary metabolites” (378, 25.1%) and “microbial metabolism in diverse environments” (326, 21.6%). The significantly enriched (corrected *p*-value < 0.05, bold in Table S8) pathway was “oxidative phosphorylation”. In addition, other enriched pathways were also observed, such as “peptidoglycan biosynthesis”, “RNA polymerase”, “ABC transporters” and “purine metabolism”.

**Figure 5:**
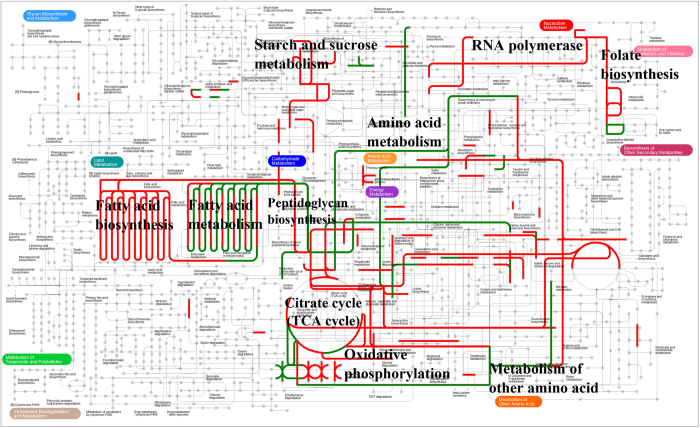
Metabolic pathways mapping of differentially expressed genes with expression fold change of at least 5-fold up- or down-regulation in the t_TG9 versus c_TG9 libraries. The red and the green lines indicate genes with up-regulated and down-regulated, respectively.

To further identify the KEGG pathways of the up- or down-regulated genes, KEGG enrichment analyses were performed on up- and down-regulated genes with the KEGG annotation, respectively. In 155 enriched KEGG pathways of up-regulated genes, the pathways of “peptidoglycan biosynthesis”, “oxidative phosphorylation” and “homologous recombination” were significantly enriched (bold in Table S9). In contrast, down-regulated genes significantly enriched in the KEGG pathways of “nitrotoluene degradation” and “degradation of aromatic compounds” (bold in Table S10). In addition, to provide a global view of metabolism of DEGs with expression fold change of at least 5-fold up- or down-regulation, 410 DEGs with different KO IDs were submitted for analysis via the on-line Interactive Pathways (ipath) explorer v2 and mapped to 16 pathways (Fig. 5). As shown in Fig. 5, the mapping pathways were related to metabolism of carbohydrates, lipid, amino acid and nucleotide, as well as vitamin synthesis. The red and the green lines indicated that the expression of genes were up- and down-regulated, respectively. The pathways, such as “citrate cycle (TCA cycle)”, “oxidative phosphorylation” and “peptidoglycan biosynthesis” showed enhancement, which were in accord with the results of enrichment analysis.

### Quantitative real-time RT-PCR (qRT-PCR) validation

**Figure 6:**
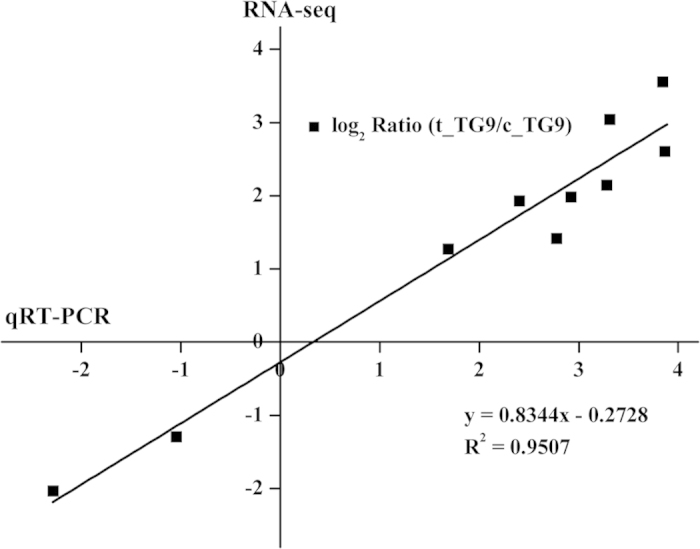
Validation of RNA-seq results by quantitative reverse transcription-polymerase chain reaction (qRT-PCR).

Ten genes with different expression profiles were randomly selected to validate the results of RNA-seq. As shown in Fig. 6, the RNA-seq data were consistent with the qRT-PCR data and Pearson correlation coefficient value was 0.975 (P < 0.0001). Although a few differences that are often found between the results of qRT-PCR and RNA-seq, up- or down-regulation of the 10 genes demonstrating in RNA-seq data were verified by qRT-PCR results (Table S11).

## Discussion

Up to now, extensive studies on the VBNC state of pathogenic bacteria, such as *V*. *cholerae*, *Vibrio vulnificus*, *Salmonella typhi*, *Escherichia coli*, *Mycobacterium tuberculosis*, *Listeria monocytogenes* and ect., have been presented^3^,^6^,^9^,^12^,^19^. Notably, it has been suggested that the maintenance of viability with loss of culturability also exists in pollutant-degrading bacteria. However, to our knowledge, there is hardly any information concerning the VBNC bacteria in contaminated environments. Furthermore, it is well-known that due to entering the VBNC state, highly efficient pollutant-degrading bacteria in the laboratory, exhibited lower efficiency and survived poorly when used in field-scale bioremediation studies^23^. Currently, enhancing activities and degrading capabilities of pollutant-degrading bacteria, and understanding their related function and genotype, are becoming more crucial^23^. Therefore, it is necessary to study the VBNC state of pollutant-degrading bacteria, which may yield insight into strategies for prevention and control the VBNC bacteria for their better capabilities in environmental rehabilitation.

In the present study, the biphenyl/PCB-degrading bacterium *R*. *biphenylivorans* TG9^T^ entered into the VBNC state after 145 days maintaining in mineral-salts medium at 4 °C. The morphological changes in VBNC cells of the strain TG9^T^ in the present study were consistent with other VBNC cells induced by nutrient-limited medium and low temperature^4^,^25^. Most of VBNC cells exhibited significant dwarfing, while some cells showed slightly elongated or non-detectable in cell size^4^,^26^. Exiting the VBNC state under favorable conditions is an important characteristic of VBNC bacteria^27^. Resuscitation from the VBNC state may be achieved by a simple reversal of the inducing factors (nutrient ability and temperature upshift)^19^,^25^, and may depend on the role of a group of extracellular bacteria proteins known as Rpfs^28^. As noted by Epstein^7^, the resuscitation process may involve “scout” cells that production and accumulation of signaling compounds resulted in the growth of the remaining dormant cells. However, some bacteria require more complex methods for resuscitation, such as a variety of environmental or chemical stimuli^29^, or co-incubation with protozoa *Acanthamoeba polyphaga*^30^. The VBNC cells of *R*. *biphenylivorans* TG9^T^ could be resuscitated after culturing on the LB agar plates at 30 °C for 3 days. For benzylpenicillin is able to kill still growing cells, benzylpenicillin pretreatment confirmed that the phenomenon of resuscitation did not attribute to the regrowth of an undetectable level of culturable cells, and the nonculturable cells were indeed in the VBNC state. The resuscitated cells, which were smaller than exponential-phase cells but larger than VBNC cells, were not in accord with that reported by Zeng *et al.*^25^ who found that there was no morphological difference between the resuscitated and normal cells. Additionally, reduction of enzymatic activity was also observed in VBNC cells of the strain TG9^T^ which led to a low level of metabolic activity. Overall, changes in morphology and enzymatic activity could be survival strategies that allowed the VBNC cells to minimize cellular maintenance requirements and ultimately adapted to hash environmental conditions^5^,^12^. Besides, Roszak *et al.*^2^ noted that microbial cell has a wide variety of genotypic and phenotypic accommodations to shift in environmental parameters.

In this work, based on the transcriptome data, the molecular levels with regard to gene expression and regulation underlying the VBNC state were uncovered. Analysis of GO functional category enrichment demonstrated that “ATP biosynthetic process”, “protein metabolic process” and “cellular metabolic process” were significantly enriched among the up-regulated genes, while “oxidation-reduction process” was significantly enriched among the down-regulated genes. Moreover, “cellular process” most highly represented in the GO terms of biological process category was in accordance with the reported by Asakura *et al.*^18^ who demonstrated that the modulated genes in the VBNC state of *V*. *cholerae* were mainly responsible for cellular processes. Meanwhile, a global view of metabolism of up- and down-regulated genes indicated that the mapping pathways of up-regulated genes were related to “starch and sucrose metabolism”, “fatty acid biosynthesis”, “ATP biosynthesis”, “peptidoglycan biosynthesis”, “folate biosynthesis” and “RNA polymerase”, while the down-regulated genes were related to “fatty acid metabolism” and “amino acid metabolism”. Combining the results of GO functions and KEGG pathways, DEGs were found to be involved in metabolism of carbohydrates, lipid, amino acid and nucleotide, as well as vitamin synthesis. In particular, these results revealed that the up-regulated genes related to ATP accumulation, protein modification, peptidoglycan biosynthesis and RNA polymerase were found in the VBNC cells, which make VBNC cells more resistant to inhospitable conditions. This is consistent with the view that ATP levels and membrane potential were generally found to still high in VBNC cells^6^,^31^. Li *et al.*^15^ demonstrated that the ATP level of the stressed *L. monocytogenes* cells was much higher than the initial ATP level of the normal cells. Accumulation of more energy in stressed cells might be helpful for recovering cells from injury and surviving longer after nutrient depletion^15^,^32^. However, it is important to point out that the changes in ATP contents varied with various stress factors and bacterial species. For instance, Stuart *et al.*^33^ reported that starvation conditions decreased the ability to synthesize ATP, which led to decreased energy consumption for promoting maintenance under adverse conditions. Similarly, decrease of respiration rates and macromolecular metabolism in VBNC cells leads to reduction of the energy requirement, which contribute to long-term maintenance of cells viability^12^,^13^. Moreover, protein modification was demonstrated to be necessary for long-term starved cells, and there is considerable evidence that these proteins might play a significant role in the induction or maintenance of the VBNC state. Asakura *et al.*^18^ demonstrated that several genes for cell envelope-associated proteins were up-regulated in the VBNC state of *V*. *cholerae*. The degradation of existing protein might be a major source of amino acids utilized for new protein synthesis during long-term starvation^34^. Furthermore, among the various wall polymers, peptidoglycan must be regarded as the main macromolecule contributing to cell shape determination and maintenance^4^. It has been demonstrated that the VBNC cells of *Enterococcus faecalis* maintain the expression of genes involved in peptidoglycan synthesis^35^, and have a hyper-cross-linked peptidoglycan compared with the peptidoglycan of normal cells. Moreover, the VBNC *E. faecalis* cells showed increasing penicillin binding proteins (PBPs 1 and 5) synthesis which are involved in the terminal stages of peptidoglycan assembly^36^. In addition, evidence has been obtained to show that ppGpp which is priming the RNA polymerase in accordance with environmental signals, is accumulated under stressful conditions^19^,^37^. Meanwhile, RNA polymerase redistributed from proliferation to maintenance occurs in harsh environments^37^.

Intriguingly, although the number of up-regulated genes was higher than that of down-regulated genes in the VBNC cells, the number of up- and down-regulated genes with expression fold change of at least 20-fold was 17 and 42, respectively. In particular, these down-regulated genes mainly encoded hypothetical protein, membrane protein and NADH dehydrogenase subunit. The results are in agreement with the GO function and KEGG pathway analysis, which indicated that down-regulated genes were mainly related to oxidation-reduction process and amino acid metabolism. It has been verified that cold-shocked cells of *V*. *vulnificus* lost catalase activity and was peroxide sensitive^31^. Besides, decreased catalase activity was observed in the VBNC state of *V. vulnificus*, which resulted in impaired detoxification of hydrogen peroxide present in routine media^38^. Furthermore, accumulation of ppGpp in the VBNC cells resulted in transcriptional shut-down of many genes involved in protein synthesis, which prevented superfluous macromolecular synthesis to minimize cell maintenance requirements^12^,^37^. In addition, it has been reported that ToxR influences the expression of more than 150 genes which involved in cellular transport, energy metabolism, motility and iron uptake, as well as the expression of outer membrane porins OmpU and OmpT in *V*. *cholerae*^16^. ToxR undergoes regulated intramembrane proteolysis (RIP) in *V*. *cholerae* in response to nutrient limitation at alkaline pH, which is dependent upon the RpoE-mediated periplasmic stress response. The loss of ToxR is associated with entry of *V*. *cholerae* into the VBNC state^39^. So entering the VBNC state is just one of adaptive response that allows bacteria to survive when conditions are not conducive to active growth. It is worth to note that the exact molecular mechanism of entry the VBNC state is yet unknown and likely differs from bacterium to bacterium^9^.

In summary, results suggest that the biphenyl/PCB-degrading bacterium *R*. *biphenylivorans* TG9^T^ could enter into the VBNC state under oligotrophic and low temperature conditions. The changes of morphology, enzymatic activity and gene expression are paramount for survival in response to harsh environmental stresses. The VBNC cells could recover culturability with simple temperature increase and nutrient addition. These findings are significant important for bioremediation of contaminated environments, where various stresses lead to lower activities of pollutant-degrading bacteria. It remains to see whether other stress conditions could induce the VBNC state of *R*. *biphenylivorans*. It can be expected that a novel efficient method might be developed to prevent and control the formation of VBNC bacteria for their better capabilities in environmental rehabilitation.

## Methods

### Bacterial strain and culture conditions

The bacterial strain *R*. *biphenylivorans* TG9^T^ (=CGMCC 1.12975^T^ = KCTC 29673^T^ = MCCC 1K00286^T^), which had previously been described^24^, was used in this study. It was stored in LB broth with 20% glycerol at −80 °C. The strain was cultured in a modified mineral-salts medium containing (per liter): 1 g KH_2_PO_4_, 3 g K_2_HPO_4_·3H_2_O, 0.2 g MgSO_4_, 0.02 g FeSO_4_·7H_2_O, 1 g NaCl, 3 g (NH_4_)_2_SO_4_, 0.01 g CaCl_2_, and biphenyl at concentration of 300 mg/L was added to the mineral-salts medium as the sole carbon and energy source. The strain was incubated at 30 °C with shaking at 180 rpm until grown to the exponential phase (OD_600_ = 0.98).

### Conditions inducing the VBNC state

Exponential-phase cells were collected by centrifuged (8000 g, 15 min) and washed twice with sterile NaCl (0.85%, w/v) solution (pH = 7.3). The washed cells were resuspended in the sterilized mineral-salts medium at a final concentration of 1 × 10^7^ CFU/mL (the control group, c_TG9). The inoculated microcosm was maintained at 4 °C for several days to induce the VBNC state (<0.1 CFU/mL) (the treatment group, t_TG9). All these experiments were performed in triplicates. Each sample obtained at distinct times was analyzed using acridine orange direct counts (AODC)^40^, flow cytometer and LB agar plates, which present total, viable and culturable numbers of bacterial cells, respectively (Fig. S1).

### Analysis of viability and culturability

Cell viability was investigated using the LIVE/DEAD *Bac*Light bacterial viability kit (Molecular Probes, Inc., Eugene, OR). Briefly, green and red fluorescent nucleic acid stains SYTO 9 and propidium iodide (PI) at a final concentration of 6 μM and 30 μM were added to each 1 mL diluted sample of cells in the BD TruCOUNT^TM^ Tubes (BD Biosciences), respectively. Samples were analyzed by an Epics XL flow cytometer (Coulter Corporation, Miami, Fla), and the fluorescence were recorded using 525 nm and 620 nm band pass filters, respectively. For assessing the culturability, each sample was serially (1:10) diluted with the sterile NaCl solution and then incubated on LB agar at 30 °C for 48 h. The culturable numbers of bacterial cells were obtained as counts per millilitre (CFU/mL) of initial suspension. All these experiments were performed in triplicates. When plate counting demonstrated a culturable cell concentration below 0.1 CFU/mL, the cells were determined to enter the VBNC state.

### Resuscitation from the VBNC state

The VBNC cells were centrifuged (8000 g, 10 min), and then resuspended in the same volume of the sterile NaCl solution. For LB liquid media, the cell suspensions were divided into two groups in resuscitation experiments. In order to exclude the possibility that the detected resuscitation was due to regrowth of still culturable cells existed in the VBNC microcosm^19^. One of the groups was supplemented with benzylpenicillin to a final concentration of 200 mg/L, and incubated at room temperature for 2 h. Then the cells were washed three times with distilled water, and resuspended in the sterile NaCl solution. for further resuscitation experiments. 10 mL of the benzylpenicillin-pretreated and untreated cell suspensions were taken from the two groups, respectively, and then their 10-fold serial dilutions in LB were transferred to 50 mL sterile glass tubes, respectively. The tubes were incubated at 30 °C in a static state for 4 days. The MPN method was used to count the resuscitated cell numbers at 12 h interval over the entire incubation time of 96 h. All the positive tubes were scored and the MPNs were calculated by using a probability table^41^. All the cultures in which visible turbidity appeared were spread onto LB plates to exclude any possible contaminated. Furthermore, the resuscitation experiments were also performed in solid media. Appropriate dilutions of benzylpenicillin-pretreated cell suspensions were spread onto LB agar plates. Then plates were incubated at 30 °C for 4 days and checked daily for the presence of colonies. All experiments described here were performed in triplicates.

### Morphology and enzymatic activity assays

Morphological characteristics of exponential-phase cells, VBNC cells and resuscitated cells were observed using confocal laser scanning microscope (CLSM 510 Meta, Zeiss) stained with SYTO 9 and PI, and scanning electron microscopy (S3000N, JEOL). The 19 different enzymatic activities of exponential-phase cells, VBNC cells and resuscitated cells were determined by an API ZYM kit (BioMérieux, France) according to the manufacturer’s instructions. Briefly, the cells were centrifuged at 8,000 g for 10 min, and then the pellets were resuspended in the sterile NaCl solution to a density approximating McFarland no. 5 or no. 6 turbidity standard. The suspensions were added to the reaction strips and incubated at 30 °C in the dark. After 4 h, colorimetric reagents were added to the reaction system. Each enzymatic activity was estimated according to the color present after reaction for 5 to 10 min.

### Illumina high-throughput transcriptome sequencing

#### RNA extraction

As shown in Fig. S1, triplicate biological samples from the c_TG9 and t_TG9 were obtained, respectively. For each biological sample, total RNA was extracted using the MICROBExpress Bacterial mRNA Enrichment kit (Life Technologies, Grand Island, NY, USA) following the manufacturer’s instructions. Then two RNA samples were randomly selected from the triplicate RNA samples, and were pooled (w/w, 1:1) as one RNA sample. Finally, this pooled RNA sample and the rest one of the triplicate RNA samples were used for transcriptome analysis. RNA was quantified by using an Agilent 2100 bioanalyzer (Agilent Technologies, Inc., Santa Clara, CA, USA). At least 20 μg of total RNA (OD260/280 = 1.8~2.2, RNA28S:18S ≥ 1.0) at a concentration of ≥ 400 ng/μL was used for cDNA library construction^42^.

#### cDNA library construction and sequencing

Illumina (San Diego, CA) sequencing was performed at the Beijing Genomic Institute (BGI-Shenzhen, China) following the manufacturer’s instructions. Briefly, rRNA was depleted using the Ribo-Zero Magnetic kit (Epicentre, Madison, WI). The mRNA was fragmented into small pieces (130-170 nt) and purified with Agencourt RNAClean XP Beads (Beckman Coulter Genomics). These cleaved mRNA fragments served as a template for the synthesis of the first-strand cDNA using First Strand Master Mix and SuperScript II reverse transcriptase (Invitrogen). Second-strand cDNA was then synthesized using SuperScript Double-Stranded cDNA Synthesis kit (Invitrogen, Camarillo, CA). These cDNA fragments were further processed with end reparation and poly (A) addition. The repaired cDNA fragments were 3’-adenylated. Illumina’s paired-end adapters were ligated to the ends of these 3’-adenylated cDNA fragments. The products from this ligation reaction were electrophoresed to select a suitable size range of fragments for PCR amplification. Finally, cDNA libraries with a fragment length range of 200 bp were constructed and sequenced on an Illumina HiSeq^TM^ 2000 (TruSeq SBS KIT-HS V3, Illumina) platform^43^.

#### Mapping reads to the reference genome

Raw reads were generated from image data and stored in FASTQ format. All the raw transcriptome data have been deposited in the NCBI Short Read Archive (SRA) database under accession number SRA221680. These raw reads were filtered to nonsense sequences including (1) reads with adaptors, (2) reads containing too many unknown base more than 5%, and (3) low quality reads which quality value lower than 10 is more than 20%. Subsequently, the clean reads were mapped to reference sequences (http://www.ncbi.nlm.nih.gov/genome/?term=NC_023150.1) using SOAPaligner/soap2^44^. Only mismatches with no more than 5 bases were allowed in the alignment. Gene coverage is calculated by the percentage of genes covered by reads.

#### Analysis of gene expression variations

To identify the DEGs between logarithmic and VBNC cells, the gene expression level is measured using RPKM method^45^. The genes that were differentially expressed between the two samples were identified using an algorithm described by Audic and Claverie^46^. The FDR ≤ 0.001 and |log2 ratio| ≥ 1 were used as the threshold to determine the statistical significant difference in gene expression^47^. For the DEGs, the GO functional analyses were performed using hypergeometric test for statistical analysis^48^. The rigorous Bonferroni correction method was used for *p* value correction. The cutoff P = 0.05 was determined after correction. GO terms fulfilling this condition were defined as significantly enriched GO terms in DEGs. In order to identify significantly enriched metabolic pathways or signal transduction pathways in which DEGs involved, the KEGG pathway enrichment analysis of DEGs was also performed^49^. After multiple testing correction, the corrected *p*-value < 0.05 was selected as a threshold to determine the significant enrichment of DEGs.

### qRT-PCR validation

To verify the RNA-seq data, 10 DEGs were randomly selected for RT-PCR. The RNA samples for RT-PCR were treated with RNase-free DNase I (TaKaRa) to remove DNA contamination. The mRNAs were extracted and transcribed to cDNAs using SuperScript III reverse transcriptase (Invitrogen) according to the manufacturer’s protocol. The cDNAs were used for qRT-PCR analysis. The qPCRs were performed in triplicates using gene specific primers (Table S1) and a power SYBR Green PCR kit (ABI). Samples were run in a MicroAmp™ 96-well reaction plate on the ABI 7500 real-time PCR system (Applied Biosystems). The normalized fold changes of the relative expression ratio were calculated using the 2^−ΔΔCT^ method^50^.

## Additional Information

**How to cite this article**: Su, X. *et al.* Identification, characterization and molecular analysis of the viable but nonculturable *Rhodococcus biphenylivorans*. *Sci. Rep.* **5**, 18590; doi: 10.1038/srep18590 (2015).

## Supplementary Material

Supplementary figures

Supplementary Table S1

Supplementary Table S2

Supplementary Table S3

Supplementary Table S4

Supplementary Table S5

Supplementary Table S6

Supplementary Table S7

Supplementary Table S8

Supplementary Table S9

Supplementary Table S10

Supplementary Table S11

## Figures and Tables

**Table 1:** An overview of the RNA-Seq statistics.

	Exponential-phase cells (c_TG9)		VBNC cells (t_TG9)	
	Number	Percentage	Number	Percentage
Total reads	13,438,262	—	12,843,362	—
Total BasePairs	1,209,443,580	—	1,155,902,580	—
Reads mapped to genome	11,731,602	87.30%	10,395,417	80.94%
Perfect match	9,748,115	72.54%	9,005,765	70.12%
≤5 bp mismatch	3,690,147	27.46%	3,850,439	29.98%
Multi-position match	740,448	5.51%	780,876	6.08%
Reads mapped to genes	7,395,075	55.03%	6,611,762	51.48%
