# Supplementary material for: Identification, characterization and molecular analysis of the viable but nonculturable Rhodococcus biphenylivorans
Source: Sci Rep. 2015 Dec 21;5:18590. doi: 10.1038/srep18590 (PMC4685647; doi:10.1038/srep18590)
Supplement: Supplementary figures [file srep18590-s1.pdf]

**Supplementary information**

# Identification, characterization and molecular analysis of the viable but nonculturable *Rhodococcus biphenylivorans*

Xiaomei Su[1,2], Faqian Sun[1,2], Yalin Wang[1,2], Muhammad Zaffar Hashmi[3], Li Guo[1,2], Linxian Ding[4], Chaofeng Shen[1,2]*

[1]Department of Environmental Engineering, College of Environmental and Resource Sciences, Zhejiang University, Hangzhou 310058, China

[2]Key Laboratory for Water Pollution Control and Environmental Safety, Zhejiang Province, China

[3]Department of Meteorology, COMSATS Institute of Information Technology, Islamabad 44000, Pakistan

[4]College of Geography and Environmental Science, Zhejiang Normal University, Jinhua 321004, China

*Corresponding author: Chaofeng Shen

Address: Yuhangtang Road 866#, Hangzhou, 310058, China

E-mail: ysxzt@zju.edu.cn; purple@zju.edu.cn

Tel: +86-571-88982016

Fax: +86-571-88982010

**Table S1.** qPCR primers used in this study.

**Table S2.** Gene expression value of annotated genes.

**Table S3.** Differentially expressed genes.

**Table S4.** Genes >20 fold up- or down-regulated in t_TG9 versus c_TG9 cultures.

**Table S5.** Gene Ontology function of differentially expressed genes.

**Table S6.** GO enrichment analysis of up-regulated genes.

**Table S7.** GO enrichment analysis of down-regulated genes.

**Table S8.** KEGG pathway enrichment of differentially expressed genes in t_TG9 and c_TG9 libraries.

**Table S9.** KEGG enrichment analysis of up-regulated genes.

**Table S10.** KEGG enrichment analysis of down-regulated genes.

**Table S11.** qRT-PCR validation of transcriptomic profiles.

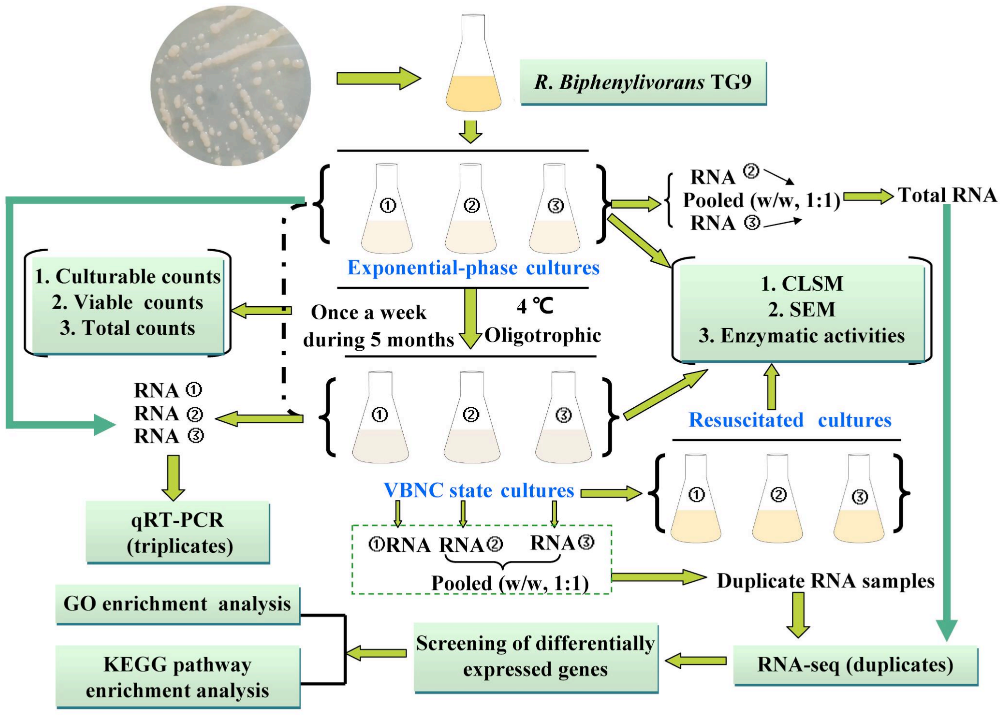


**Fig. S1.** Schematic flow diagram of the experimental design.

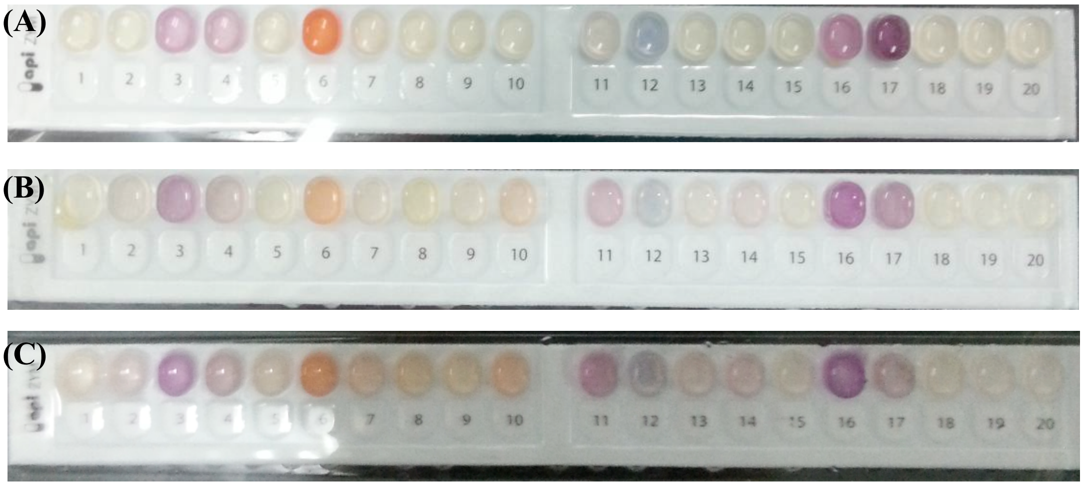


**Fig. S2.** Enzymatic activities of *Rhodococcus biphenylivorans* TG9$^T$ measured by the API ZYM system. (A) exponential-phase cells; (B) VBNC cells; (C) resuscitated cells.
